# Monitoring and Assessing the Degradation Rate of Magnesium-Based Artificial Bone In Vitro Using a Wireless Magnetoelastic Sensor

**DOI:** 10.3390/s18093066

**Published:** 2018-09-12

**Authors:** Limin Ren, Kun Yu, Yisong Tan

**Affiliations:** School of Mechanical Engineering, Northeast Electric Power University, Jilin 132012, China; renlimin@neepu.edu.cn (L.R.); 2201700350@neepu.edu.cn (K.Y.)

**Keywords:** magnesium-based artificial bone, magnetoelastic sensor, wireless and passive, degradation rate

## Abstract

A magnetoelastic-based (MB) sensor was employed as a novel method to monitor and assess the degradation rate of magnesium-based artificial bone (MBAB) in vitro, which can be used as an implant to repair a bone defect, providing a quantitative method to depict the degradation rate of MBAB. MBABs were fabricated by the Pro/Engineering software and a precision machine tool using high-purity (HP) magnesium. The MB sensor was embedded in the neutral surface of MBAB by an unharmful quick adhesive, forming the MB sensor-embedded MBAB (EMBAB). The modified simulated body fluid (MSBF) media (PH = 7.4), mimicking the human internal environment, and the NaOH media (PH = 12), accelerating EMBAB’s degradation, were used to immerse the EMBAB for 15 days at 37 °C. The EMBAB was then tested daily on a self-developed experimental platform to monitor the relative output power under a 100 N external force. The results showed that the relative output power of the sensing coil gradually increased with the EMBAB’s degradation. The degradation rate of the EMBAB could be calculated on the basis of the changes of the relative output power caused by the MB sensor and of the degradation time. With the EMBAB’s degradation, an increasing strain directly worked on the MB sensor, significantly changing the value of the relative output power, which means that the EMBAB was characterized by a quick degradation rate. During the 15 days of the experiment, the degradation rates on the 7th and 15th days were 0.005 dbm/day and 0.02 dbm/day, and 0.02 dbm/day and 0.04 dbm/day in MSBF and alkaline media, respectively. Therefore, the MB sensor provides a wireless and passive method to monitor and assess the degradation rate of bone implants in vitro.

## 1. Introduction

Biodegradable artificial bones have been used as an alternative biomedical apparatus for the internal repair of human bone defects, particularly load-bearing bone defects [1,2,3,4,5,6]. Biodegradable bone defect repair devices provide several advantages. First, there is no need to remove the devices after the bone defect heals, as is the case for metal fixation devices [7]. Second, using bioabsorbable implants prevents the stress-shielding atrophy and weakening of the fixed bone that is usually caused by rigid metallic fixation [8]. The ability to control the degradation rate of an artificial bone is critical to the success of its application. However, the prolonged presence of the artificial bone can interfere with the integration of the new bone that forms during healing. Thus, the rate of degradation of biodegradable artificial bone must be tailored to match the rate of new bone ingrowth as the bone heals.

Magnesium and its alloys are often used in implants and as replacements of human bone to repair defects or fractures [9,10,11] because they exhibit biocompatibility and appropriate mechanical properties [12]. Importantly, the elastic modulus of suitable magnesium alloys is about 40–50 GPa, which is very close to that of human bone (10–40 GPa). Hence, these alloys can also minimize the stress-shielding phenomena caused by other metallic implants made of stainless steel or titanium alloys [13]. The other metallic biomaterials are essentially neutral in vivo, remaining as permanent fixtures, which, in the case of plates, screws, and pins used to secure serious fractures, must be removed by a second surgical procedure after the tissue has healed sufficiently [14]. Repeated surgery increases both costs for the healthcare systems and patients’ morbidity [15]. Conversely, Mg and Mg alloys can degrade completely under physiological conditions, avoiding the need for a second surgical intervention to remove the implant after bone healing. 

The PH of subcutaneous tissues (PH = 6.7–7.1) [16], tumor tissues (PH < 6.9) [17], and internal tissues after prolonged hemorrhage (PH < 7) [18,19] are different. Therefore, there is a need to characterize the degradation rate of biodegradable artificial bone under multiple conditions to better mimic various physiological environments. This requires a significant number of experimental settings. Nowadays, the characterization of the degradation behavior of artificial bone still involves tracking its mass loss over time [20,21], which requires large amounts of samples. Because of the need for a large quantity of material in traditional degradation testing, comprehensive studies to evaluate the effects of multiple factors on the degradation behavior and rate of artificial bone are cost-prohibitive. Currently, no accurate methods exist for quantitatively monitoring the in vivo biodegradation behavior and rate of artificial bone.

Recently, the authors of this paper have reported the use of a magnetoelastic-based (MB) sensor to monitor a bone plate strain over time [22]. The MB sensor is made of magnetoelastic material, such as Metglas 2826MB (Fe_40_Ni_38_Mo_4_B_18_). Because of its large magnetoelastic coupling factor (~0.98) and a Magnetostriction in the order of 10^−5^ [23,24], the Metglas-based sensor exhibits vibrations when excited by a magnetic AC field. At the resonant frequency of the MB sensor, the vibration also generates a significant magnetic field that can be remotely detected with a coil antenna [25]. When a mass is applied on the sensor surface, it causes a change in the relative output power of the sensing coil. In addition, the change of the relative output power caused by the sensor depends on the elasticity of the applied coating or the viscosity of its surrounding medium. The ability to wirelessly monitor a change in mass, elasticity, viscosity, and force enables the MB sensor to detect the viscosity of chemical and biological agents [26,27] and other materials [28]. Specifically, with proper surface functionalization, the MB sensor can be used in cell culture or even implanted in vivo to monitor biointerfacial binding events, such as cellular attachment and proliferation [29]. Remote query capability and the long-term durability of a functionalized MB sensor in a biological environment mean the sensor is suitable for monitoring artificial bone degradation in real time. Furthermore, compared to the traditional methods, the MB sensor also requires a significantly lower sample volume for testing.

A wireless and passive magnetoelastic-based sensor is therefore proposed in this work to monitor the degradation rate of magnesium-based artificial bone over 15 days in vitro. The MB sensor was embedded inside a magnesium-based artificial bone (MBAB) mimicking the human natural bone. In this study, the MBAB was designed and fabricated by the Pro/Engineering software and a precision machine tool using high-purity (HP) magnesium. The MB sensor was embedded inside the MBAB with a quick adhesive, forming the embedded MBAB (EMBAB). The modified simulated body fluid (MSBF) media (PH = 7.4) and the NaOH media (PH = 12) were used to immerse the EMBAB, and then the performance of the MB sensor was tested with a wireless and passive method to monitor the changes in the relative output power, which can be used to calculate the degradation rate of the EMBAB incubated at 37 °C to achieve thermal equilibrium.

## 2. Materials and Methods

### 2.1. MB Sensor Working Principle

**Figure 1:**
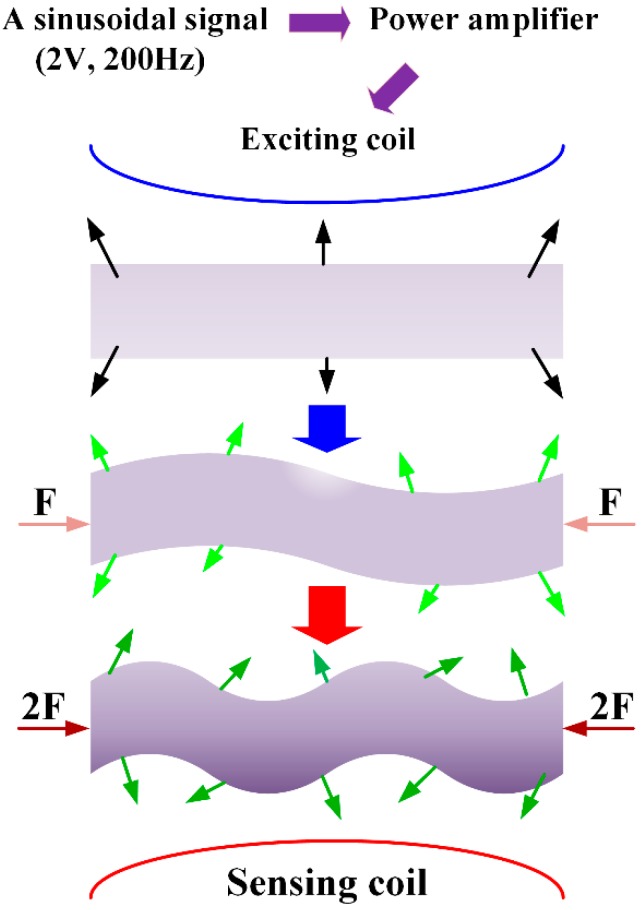
Working principle of the magnetoelastic-based (MB) sensor (working condition of MB sensor under F = 50 N and 2F = 100 N).

Magnetoelastic material (Metglas 2826MB, Hong Kong, China) with 40% Fe, 38% Ni, 18% B, and 4% Mo was used as the MB sensor in this paper to monitor and assess the degradation rate of the EMBAB, which is a ribbon-like amorphous ferromagnetic alloy. An MB sensor characterized by low resistivity, high permeability, and a high magnetoelastic coefficient was chosen for the following analyses [25,30,31,32,33]. Figure 1 shows the working principle of the MB sensor. A sinusoidal signal with a frequency of 200 Hz and a voltage of 2 V is generated by a function generator. The sinusoidal signal is amplified by a power amplifier and then is input into the exciting coil, which generates an alternating magnetic field. When the MB sensor is subject to tension or compression strain under an external magnetic field, the permeability of the MB sensor will change accordingly. The variation of the permeability can cause a magnetization change in the magnetic field, which is detected by the sensing coil [34]. Therefore, the variation of the external magnetic 100 field can reflect the applied strain. This is the inverse magnetoelastic effect (Villari effect). In this study, the degradation of the EMBAB caused a change of strain on the MB sensor. Hence, the MB sensor could be used to monitor the degradation behavior of the EMBAB with a wireless and passive method. In fact, during this process, no cables or batteries were needed for the MB sensor.

### 2.2. Preparing the EMBAB

**Figure 2:**
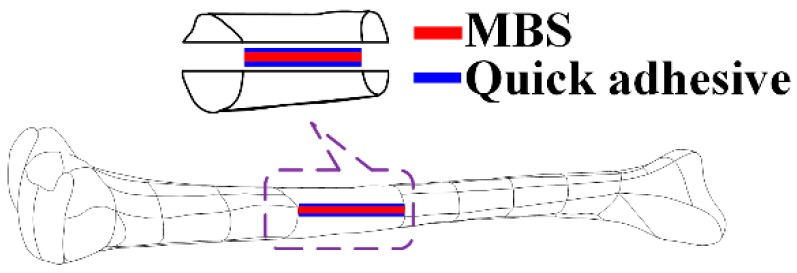
Model of the embedded magnesium-based artificial bone (EMBAB). MBS: magnetoelastic-based sensor.

The high-purity magnesium [1] (99.99 wt.% Mg; 0.002 wt.% Si; 0.0015 wt.% Fe; 0.0008 wt.% Mn; 0.0002 wt.% Ni; 0.0003 wt.% Cu) used in this experiment to fabricate the MBAB was supplied by Suzhou Origin Medical Technology Co., Ltd., Suzhou, China. The 3D defect models of human bone, split in two parts from the neutral surface, were firstly designed by the Pro/Engineering 4.0 software based on the data from the FreeScan X7 Hand-held 3D scanner of Beijing Tianyuan 3D technology Co., Ltd., Beijing, China. The 3D models were then converted to the G-code one by one using the Computer-Aided X Alliances software (CAXA, Version 2015, Beijing, China) that can be recognized by the miller FF 500 CNC of Proxxon, PROXXON GmbH, Dieselstraße 3–7, Germany. Subsequently, the CNC, was used to automatically machine the MBABs (25 mm of length with an irregular external surface). The MB sensor was sonicated in ethanol for five minutes, rinsed with deionized water, and dried. Then, the MB sensor (20 mm × 10 mm × 30 μm) was embedded in the fractured surface of the MBAB with the 302 modified acrylate quick adhesive (Gelianghao New Material Co., Ltd., Shenyang, China) to fabricate the EMBAB, as shown in Figure 2, which was dried and stored under vacuum until use. 

The presence of the EMBAB was verified by determining the change of relative output power before and after the immersion process. The quick adhesive used is not harmful for the human body, as indicated by the Food and Drug Administration (FDA) in the U.S. and the CE Marking in Europe [35,36], which forbid the utilization of unsafe adhesives in humans.

### 2.3. Monitoring and Assessing the Degradation Rate of the EMBAB

**Figure 3:**
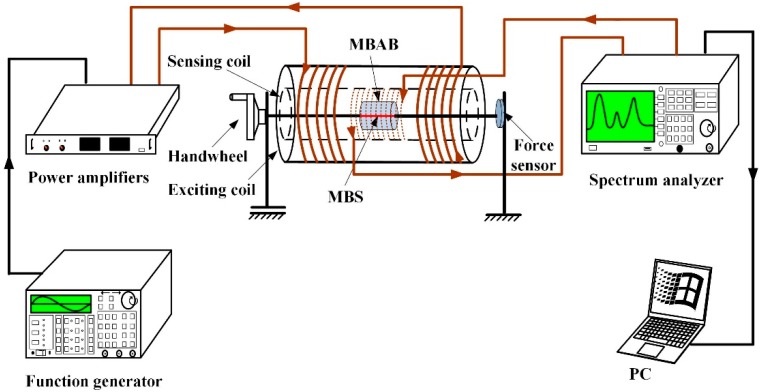
Experimental platform and devices. The EMBAB is fixed in the middle of the platform by two stainless steel bars surrounding the sensing coil (light brown dotted line) and the exciting coil (thick brown line). The external force was kept at 100 N in each daily test.

Figure 3 shows the graphical representation of the experiment. The devices and platform shown were used to assess the degradation rate of the EMBAB daily after the EMBAB was immersed in the MSBF and the NaOH media. The EMBAB was fixed well in the inner part of the sensing coil (200 turns, 110 mm in length, 0.5 mm in diameter) and exciting coil (200 turns, 90 mm in length, and 0.25 mm in diameter) by two stainless steel bars installed in the experimental platform. A sinusoidal source (2 V, peak to peak, 200 Hz) generated by a function generator (Fluke 271, Fluke Corporation. 6920 Seaway Boulevard Everett, WA, USA) and amplified by a power amplifier (TAPCO JuiceTM, LOUD Technologies Inc., Woodinville, WA, USA), was input into the exciting coil to generate an alternating magnetic field. The MB sensor is affected by the strain produced by the external force (100 N) in the alternating magnetic field and produces an inverse magnetostrictive effect (Villari effect), which leads to a permeability variation of the MB sensor and can result in a change of the spatial magnetic field. The sensing coil was connected to a spectrum analyzer (GA40XX, Guorui Antai technology Co., Ltd., Nanjing, China) to detect wirelessly the change of the spatial magnetic field, which was indicated by the relative output power of the sensing coil. The change of the relative output power caused by the MB sensor increased with the degradation of the EMBAB, which means that the EMBAB’s degradation was associated with an increased strain on the MB sensor. In fact, the external force worked directly on the MB sensor causing a stronger strain as the degradation of the EMBAB proceeded. Therefore, the MB sensor can reflect and monitor the degradation behavior and rate of the EMBAB through a wireless and passive method. 

A great advantage of this technique to assess the degradation rate of an MBAB is that, in contrast to the traditional methods [4,13,37], it does not require any additional operations once the MB sensor is embedded into the MBAB. Therefore, the MB sensor is a convenient and preferable sensing element for the passive and wireless method to assess the degradation rate of an EMBAB. Importantly, during the degradation period, the MB sensor can induce different output powers corresponding to the progressive EMBAB degradation in time. Hence, the degradation rate of an EMBAB (*Y*) can be determined considering the time (e.g., days) since the experiment’s start (*T_n_* = 1, 2,…,15) and the varying value of the relative output power (*Q_n_*), following the Equation (1):

(1) $$Y(\mathrm{dbm}/\mathrm{day})=\frac{Q_{n}}{T_{n}}\;$$

*Q_n_* is the varying value of the relative power output at day n, and *T_n_* = 1, 2, …, 15 is the time at which the degradation is measured. All data are presented as the mean value ± standard deviation (SD). Statistical comparisons were performed using one-way ANOVA, and *p* < 0.05 was considered significant.

After initial characterization, the EMBABs were placed in 2 mL vials containing MSBF and basic NaOH media for 15 days, and then were incubated at 37 °C to achieve thermal equilibrium. The degradation media were renewed throughout the degradation period every 24 h. The immersion media for the EMBABs were prepared according to Oyane et al. [38]. The ion concentration in the MSBF was 142 mM for Na^+^, 5.0 mM for K^+^, 1.5 mM for Mg^2+^, 2.5 mM for Ca^2+^, 103 mM for Cl^−^ 10.0 mM for HCO^−3^, 1.0 mM for HPO2^−4^, and 0.05 mM for SO2^−4^ [39]. Two EMBABs were prepared for each ph. During the degradation process, each EMBAB was analyzed two times a day using the described experimental platform and devices to track its degradation. The degradation rate of the EMBAB was calculated by Equation (1).

## 3. Results

### 3.1. Experimental Results

**Figure 4:**
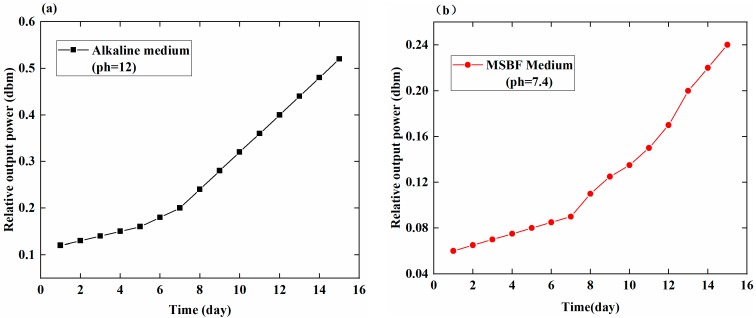
Relationship between relative output power (dbm) and time (days) using the MB sensor to assess the degradation state of the EMBAB in different media. (**a**) Relative output power of EMBAB in the alkaline medium over 15 days. (**b**) Relative output power of EMBAB in the MSBF medium over 15 days.

Figure 4 shows the relationship between the time (day) and the relative output power (dbm) of the sensing coil that represents the trend of the EMBAB degradation in the MSBF and alkaline media, respectively. Figure 4a shows the EMBAB’s degradation rate in the alkaline medium over 15 days. At the beginning, the relative output power is about 0.1 dbm. In the following days, the relative output power increases, reaching about 0.53 dbm after 15 days. Importantly, the relative output power in the first 10 days approaches the value of 0.32 dbm. Figure 4b shows that in the MSBF media, the relative output power of the EMBAB increases nonlinearly with the time, differently from what observed in the alkaline medium (Figure 4a). The value of the relative output power is 0.18 dbm after the 15 days of degradation. The alkaline medium can accelerate the degradation rate of the EMBAB and it also generates Mg(OH)_2_ that can affect the PH of the medium; Also the MSBF medium gives rise to Mg(OH)_2_. Therefore, it is important, for accurate experimental results, to check the PH of the alkaline and the MSBF media every 24 h.

The MB sensor can assess the degradation rate of the EMBAB as an implant on the basis of the different relative output powers in time. Under the same external environment, the degradation rate of the EMBAB is only influenced by the media, which is kept at a certain PH (12 and 7.4), and all other effects are eliminated. Thus, the accurate degradation rate of the EMBAB is determined to a large extent by the changing value of the relative output power induced by the MB sensor.

**Figure 5:**
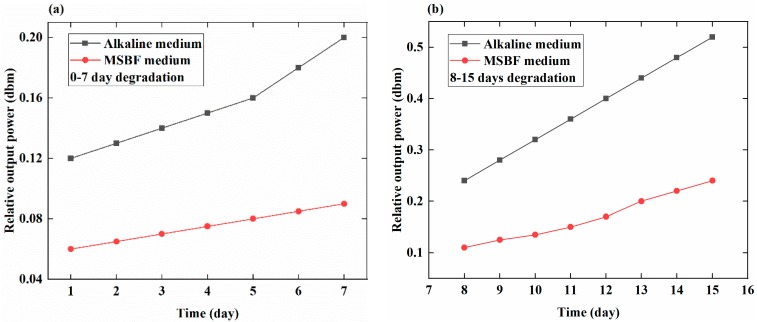
Comparison of the relative output power at different times in alkaline (PH = 12) and MSBF media (PH = 7.4). (**a**) Degradation during 0–7 days; (**b**) degradation during 7–15 days.

Figure 5 shows the relationship between the relative output power (dbm) and time (days) for the different media (alkaline medium PH = 12 and MSBF medium PH = 7.4) in the first seven days and in the next seven days, separately. In the first week, the value of the relative output power in the alkaline medium changes of about 0.08 dbm, whereas in the MSBF medium it changes of about 0.03 dbm. This different range can reflect the different degradation rate of the EMBAB in the two media. As a result, the calculated degradation rates of the EMBAB in the alkaline and MSBF media after seven days are 0.011 dbm/day and 0.0043 dbm/day, respectively, based on Equation (1). The degradation rates in the two media are comparable, which indicates the low of degradation tendency of the EMBAB. In the second week (days 8–15), the relative output power of the EMBAB in the alkaline and MSBF media changes of 0.3 dbm and 0.12 dbm, respectively, and the corresponding degradation rates are 0.043 dbm/day and 0.017 dbm/day, based on Equation (1). The degradation rate in the second week is thus larger than that of the first seven days because of the much greater strain on the MB sensor, which produces a larger degradation rate.

### 3.2. Degradation Rate of the EMBAB

**Figure 6:**
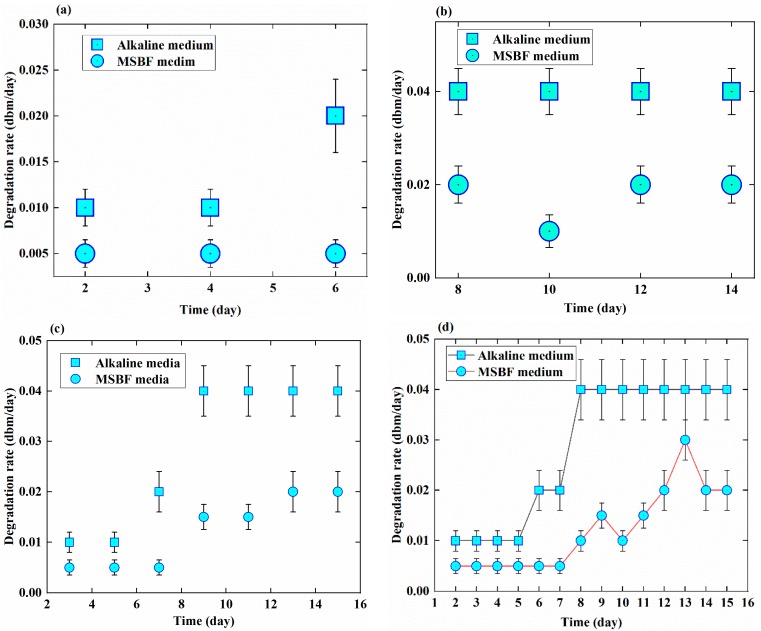
Degradation rate of the EMBAB at different times in alkaline and MSBF media. The graphs show: (**a**,**b**) degradation rate on the even number days; (**c**) degradation rate on odd number days; (**d**) degradation rate over 15 days.

Figure 6 displays the degradation rate of the EMBAB on every other day, starting from day 2. A similar trend of the EMBAB degradation is clearly evident in both media on the 2nd and 4th day, and in MSBF medium only on the 6th day; the degradation rate of the EMBAB is 0.005 dbm/day on days 2 and 4 in the alkaline medium and 0.01 dbm/day on days 2, 4, and 6 in MSBF medium, but it reaches 0.20 dbm/day on the 6th day in the alkaline medium. The degradation rates of the EMBAB remain stable at 0.04 dbm/day and 0.02 dbm/day in the alkaline medium and in MSBF, respectively, on the 8th, 10th, 12th, and 14th days. As shown in Figure 6c, in the odd number days, the degradation rate maintains trends are similar to those in Figure 6a, b. As a result, the degradation rate of the EMBAB over 15 days is quicker in the alkaline medium than in the MSBF medium, as shown in Figure 6d.

## 4. Discussion

In this study, a novel method employing a magnetoelastic-based sensor, is applied to monitor and assess the degradation rate of an MBAB, which was designed and made by using HP magnesium and a quick adhesive. EMBABs were immersed in MSBF and alkaline media and were monitored by the MB sensor to record their relative output powers (dbm) daily, as shown in Figure 4 and Figure 5 which indicate the relationship between time (days) and the relative output power (dbm) under the same environmental conditions (applied external force of 100 N). It was shown that, in the different media, the degradation degree of the EMBAB can be expressed by the change in the value of the relative output power induced by the MB sensor.

Figure 4 clearly shows the degradation state of the EMBAB. During the experiment time, the thickness of the EMBAB gradually reduced, which caused an increasing strain produced by the external force (100 N) on the MB sensor. In the initial days, the small value of the relative output power was due to the small strain applied on the MB sensor. In the following days, the degradation degree of the MBAB increased and the relative output power rose accordingly, for much more strain directly worked on the MB sensor. Figure 4 shows a low value and the following with a higher value of degradation. The reason is that the thickness of the EMBAB reduced slowly in the beginning and a little strain was applied on the MB sensor by the external force (100 N). Hence, it caused a small value of the relative output power, as shown in the 0–7 days of Figure 4. In the following days (7–15 days) with the increasing of the degradation time, the external surface of the EMBAB was gradually corroded, which led to the alteration of the EMBAB thickness and the degradation degree of the MBAB. And much more strain was exerted on the MB sensor by the external force. It caused the relative output power to rise quickly. The larger the strain working on the MB sensor, the greater the degradation rate of the EMBAB, shown in days 8–15 of Figure 4. This phenomenon indicates that the MB sensor can monitor the degradation rate of an EMBAB and thus provide a quantitative method directly describing the degradation state of an MBAB in vitro. 

During the whole degradation process of the EMBAB, the degradation rate was assessed on the basis of the changes in the values of the relative output power of the sensing coil, which were caused by the strain working on the MB sensor. In fact, with the increasing of the degradation time, the external surface of the EMBAB was gradually corroded, which led to the alteration of the EMBAB thickness. In this way, an increasing strain was produced by the external force working on the MB sensor, which caused the EMBAB degradation. In other words, the larger the strain working on the MB sensor, the greater the degradation rate of the EMBAB. Therefore, in this paper, the strain working on the MB sensor is proportional to the degradation rate of the EMBAB.

The change in the value of the relative output power in the initial days of degradation was small because of the small strain produced by the external force on the MB sensor. Compared with the alkaline medium, the limited degradation of the EMBAB in the MSBF medium could not cause a large strain on the MB sensor; therefore, the change in the value of the relative output power in the MSBF medium was small. During the whole degradation period, the value of the relative output changed by 0.41 dbm in the alkaline medium and by 0.18 dbm in the MSBF medium; the corresponding average degradation rates over 15 days were 0.027 dbm/day and 0.012 dbm/day. 

The results of Figure 6 show that the EMBAB itself resisted the external force that could not directly work on the MB sensor. However, in the next week, much more strain was applied on the MB sensor which caused a higher relative output power increasing with time, as shown in Figure 6b. Importantly, these data further demonstrate that the degradation rate of the EMBAB can be monitored and assessed by the MB sensor. Over the 15 days of the experiment, the degradation rate presented an increasing trend in both media, which means that if the EMBAB is implanted in the human body, the MB sensor can be used to monitor and assess its degradation state and rate from the moment of implantation until it is fully degraded.

**Figure 7:**
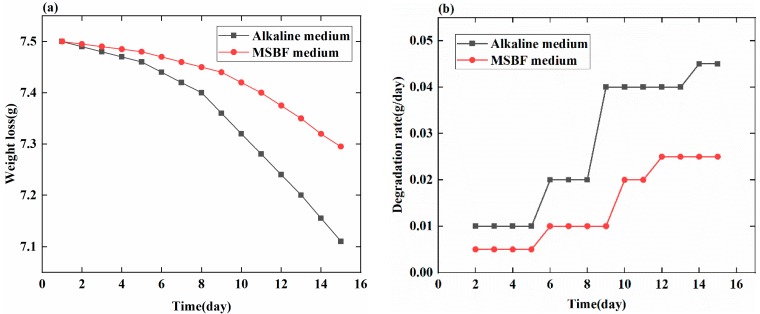
Weight loss (g) and degradation rate (g/day) of MBAB with increasing degradation time in alkaline medium and MSBF medium. (**a**) Weight loss of EMBAB in the alkaline and MSBF medium over 15 days. (**b**) Degradation rate of EMBAB in the alkaline and MSBF medium over 15 days.

To measure the degradation rates of magnesium alloys, two traditional techniques [40] are usually employed, namely the weight loss method [41] and the hydrogen evolution method [42]. The MB sensor is used to monitor and assess the degradation rate of magnesium-based artificial bone (MBAB) for the first time. Therefore, the weight loss is used as a traditional method to assess the degradation rate of MBAB and further verify the availability of MB sensor. The *X* (g/day) refers to the degradation rate that is calculated by the weight loss of MBAB. Importantly, if the values of *X* and *Y* have similarly varying trends, it indicates that the *Y* (dbm/day) to some extent can judge the change of the sensor that is caused by the degradation. The MBABs are immersed in the alkaline medium and MSBF medium for 15 days and weighed with a balance every day after the surface liquid is removed with a paper towel (Figure 7a). The *X* (g/day) refers to the degradation rate that is calculated by the weight loss of MBAB and the time, shown in Figure 7b.

(2) $$X(g/\mathrm{day})=\frac{M_{n}}{T_{n}}\;$$

*M_n_* is the varying value of the mass of the MBAB at day *n*, and *T_n_* = 1, 2, …, 15 is the time between two measuring instants. The tendency of weight loss of the MBAB shown in Figure 7a is similar to that in the literature [43], which uses the weight loss of magnesium alloy to judge the degradation. The changing trend of degradation rate *X* (g/day) calculated by the weight loss shown in Figure 7b is analogous to the *Y* (dbm/day) figured out by the relative output power of the MB sensor. Therefore, the relative output power of the MB sensor can be used to monitor and assess the degradation rate of magnesium-based artificial bone (MBAB).

In future work, we plan to use the MB sensor to monitor and assess MBAB degradation in vivo. The degradation of Mg and its alloys as biomaterials has been examined with a variety of methods in several works. Zhang et al. [44] and Wong et al. [41] focused on the mass loss to judge the degradation condition of magnesium in vitro. This is a simple and low-cost method to measure the degradation state of a biomaterial, but it needs multiple samples for accuracy and cannot supply information on how corrosion proceeds with time. Bender S [45] and Song G [46] indicated that magnesium reacts with water to form hydrogen, which means that 1 mol of magnesium produces 1 mol of hydrogen. Hence, these methods measure hydrogen evolution to determine the degradation state of magnesium. However, continuous measurements are difficult to make (i.e., capture H_2_), and a large number of influencing factors must be considered during the setup and running of a test, which can largely impact the results, leading to their irreproducibility. Monitoring of the PH has been used widely in the Mg biomaterial literature [47,48,49]. One issue, however, is that the bulk PH may not be representative of the PH of the sample’s surface and may vary by several PH units [50]. Nevertheless, the MBAB-covered MB sensor presented in this paper could be easily used to monitor and assess the MBAB degradation state in the absence of interfering factors, such as mass loss, hydrogen evolution measurements, and PH monitoring. Meanwhile, the highly sensitive MB sensor is also cost-effective and easy to operate for broad clinical applications [22,28].

Although the potential of magnesium and its alloys for biological applications is clear, the development of a clinically relevant biomedical magnesium implant would require thorough in vivo testing, initially using animal models and eventually humans. However, several factors significantly hinder the effective use of in vivo tests, including cost and time, and, most notably, the potential harm and discomfort that such studies can cause to the experimental subjects. Thus, it is vital to use appropriate in vitro tests to pre-screen Mg alloys to determine their suitability for subsequent in vivo studies. The remote sensing technology reported in this paper can potentially be further engineered to track the degradation rate of magnesium-based biomaterials in vivo. MB sensors have previously been utilized to characterize biointerfacial events in animal models [29]. However, to implement this technology in vivo, numerous challenges must be solved. One of these is the need to distinguish sensor responses associated with MBAB degradation from those associated with the inflammatory response, dynamic motions, and mechanical forces present at the implantation site.

## 5. Conclusions

Magnesium and its alloys as degradable biomaterials have been extensively applied to repair bone defects or fractures. A magnesium-based artificial bone (MBAB) defect model was designed and fabricated with the Pro/Engineering software and a precision machine tool, and then a magnetoelastic-based (MB) sensor was embedded in the neutral surface of the MBAB, thus forming the EMBAB. Importantly, a novel method based on the MB sensor was employed to monitor and assess the degradation rate of the MBAB, using a self-developed experimental platform and devices. The MSBF medium (PH = 7.4) and NaOH medium (PH = 12) were used to immerse the EMBAB over 15 days, during which period the EMBAB was tested daily by the MB sensor to measure the relative output power. The results showed that the relative output power gradually increased with time, under a 100 N external force, indicating increased degradation. The degradation rate was determined by the changes in the value of the relative output power over time. Therefore, the degradation rate of an EMBAB can be clearly monitored and assessed by the MB sensor. Over 15 days, the average degradation rates were 0.027 dbm/day and 0.012 dbm/day in alkaline and MSBF media, respectively. The degradation rate on each single day could also be calculated. In future work, the EMBAB will be implanted in animals to repair a bone defect and the MB sensor will be used to monitor and assess the degradation rate of the EMBAB in vivo.
